# Pre‐Refinement Prediction of Dopant Site Occupancy in M‐Type Hexaferrites via Hierarchical and Uncertainty‐Aware Machine‐Learning

**DOI:** 10.1002/advs.78493

**Published:** 2026-10-29

**Authors:** Dan Liu, Jianyu Ren, Ruoshui Liu, Yinong Yin, Jianfeng Xi, Xin Ming, Jiazheng Hao, Tongyun Zhao, Fengxia Hu, Baogen Shen

**Affiliations:** ^1^ Department of Physics Beijing Technology and Business University Beijing China; ^2^ Ningbo Institute of Materials Technology and Engineering Chinese Academy of Sciences Ningbo China; ^3^ Feicui Lake Laboratory School of Materials Science and Engineering Anhui University Hefei China; ^4^ Spallation Neutron Source Science Center (SNSSC) Dongguan China; ^5^ State Key Laboratory of Magnetism Institute of Physics Chinese Academy of Sciences Beijing China

**Keywords:** dopant occupancy, machine‐learning, M‐type hexaferrites, structural refinement

## Abstract

Dopant site occupancy bridges chemical composition, local crystal structure, and macroscopic functional responses in M‐type hexaferrites. However, reliable occupancy hypotheses are difficult to establish before structural refinement. Here, we develop a pre‐refinement machine‐learning framework using 494 dopant‐level records extracted from refined structures. This workflow combines Ba/Sr‐vs.‐Fe sublattice classification, fractional occupancy prediction over five inequivalent Fe sites, and reliability‐aware assessment. The CatBoost classifier achieves 96% accuracy on external validation for sublattice assignment. For Fe‐sublattice dopants, homogeneous occupancy‐model screening and heterogeneous cooperative occupancy‐model screening yield a macro mean absolute error as low as 0.0584, while preserving dominant‐site identification and occupancy ranking. The results show that dopant site occupancy is jointly controlled by dopant chemistry, target‐dopant amount, and co‐doping environment rather than a universal site‐preference rule. By integrating TabPFN baseline comparison, training‐experience assessment, and model‐consistency analysis, the framework identifies boundary and extrapolative samples that require priority refinement. Our predictions for W‐doped and Sc‐doped M‐type hexaferrites successfully reproduce key experimental occupancy trends. This work offers a practical data‐driven strategy to generate physically plausible occupancy hypotheses and assign refinement priorities for partially occupied oxide systems.

## Graphical abstract

Refined data are extracted from CIF files to construct a hierarchical machine‐learning framework integrating sublattice classification, Fe‐site occupancy prediction, and reliability assessment. External validation yields 96% accuracy for sublattice classification and a macro mean absolute error of 0.0584 for Fe‐site prediction. This study provides a basis for initial occupancy hypotheses and refinement prioritization in M‐type hexaferrites.

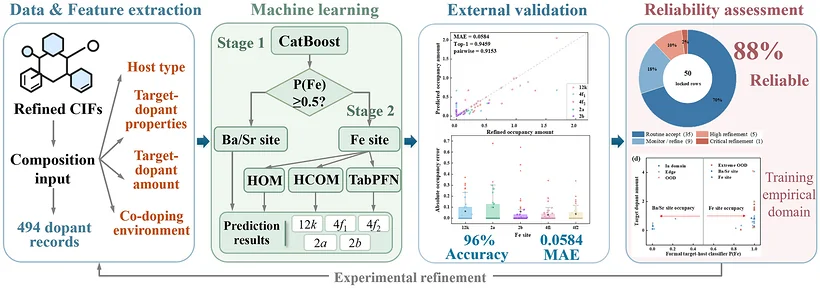

## 1 Introduction

M‐type hexaferrites, represented by BaFe_12​_O_19_​ and SrFe_12_​O_19_​, are among the most extensively studied ferrimagnetic oxides because of their high magnetocrystalline anisotropy, good chemical stability, and tunable electromagnetic responses [1, 2, 3]. Owing to these characteristics, they have long been considered important in permanent magnets, microwave and millimeter‐wave devices, electromagnetic absorption, magnetic recording, and high‐frequency functional components. Elemental doping is one of the most effective approaches for tailoring magnetic anisotropy, coercivity, saturation magnetization, dielectric response, and spin‐related interactions. However, the effect of doping is not determined solely by chemical composition. A decisive factor is where the dopant enters the crystal structure.

The magnetoplumbite structure of M‐type hexaferrites (space group P6_3_/mmc) contains one alkaline‐earth sublattice occupied by Ba or Sr and five inequivalent Fe sites, namely 12*k*, 4*f*_1_​, 4*f*_2_, 2*a*, and 2*b*. These Fe sites differ in local coordination geometry, multiplicity, and magnetic role in the superexchange network [1, 4]. Such occupancy behavior directly affects local coordination environments, Fe‐O‐Fe exchange pathways, hyperfine interactions, and ultimately the macroscopic magnetic and electromagnetic properties. Therefore, dopant site occupancy is not merely an auxiliary structural detail, but rather a critical bridge linking chemical composition, local structure, and property regulation.

Although the site occupancy of dopant elements is crucial, its determination remains challenging. In practice, site assignment is commonly inferred through a combination of Rietveld refinement [5], Mössbauer spectroscopy, neutron diffraction [6], local structural characterization, and crystal‐chemical reasoning. These approaches are indispensable and physically grounded, yet they become increasingly laborious when the system involves low dopant concentration, weak occupancy contrast, partial occupancy, multi‐site competition, or multi‐element co‐doping. In many realistic cases, occupancy determination still depends on repeated trial‐and‐error refinement guided by prior experience. This process is time‐consuming and often hard to generalize across a broad chemical space, especially when one aims to screen many candidate dopants or co‐doping schemes at the early stage of materials design.

Recent advances in materials machine‐learning (ML) have expanded data‐driven research from property prediction to crystal generation, candidate screening, and refinement‐assisted structure analysis [7, 8, 9, 10]. As a large‐scale screening framework, GNoME integrates graph neural networks, active learning strategies, and density functional theory (DFT) validation, enabling identification of thermodynamically stable crystalline candidates across diverse chemical spaces [11, 12]. Benefiting from generative artificial intelligence, the ML models have entered the stage of active periodic crystal structure design. Specifically, MatterGen realizes conditional inorganic material generation constrained by chemical composition, crystallographic symmetry, and target functional properties [13], while DiffCSP achieves coupled generation of lattice parameters and fractional coordinates under periodic E(3)‐equivariant geometric constraints. Li et al. developed the PXRDGen architecture to bridge powder x‐ray diffraction representation, conditional crystal generation, and Rietveld refinement, which proves the feasibility of embedding ML algorithms into powder diffraction structure solution and refinement pipelines [14]. To enable structure generation under crystallographic constraints, the space group and Wyckoff sites were adopted as the means to describe the crystal structure [15, 16]. Collectively, these methodological improvements empower data‐driven models to decipher hierarchical crystallographic information, breaking through the limitation of traditional composition‐property correlation modeling. Nevertheless, crystallographic research does not aim to replace conventional refinement or advanced characterization techniques via ML tools. Instead, ML models are expected to provide credible site‐occupancy hypotheses before crystallographic refinement. Ideally, a qualified pre‐refinement generative model should output probabilistic site occupancy schemes that conform to practical fractional occupancy characteristics, quantify prediction confidence, and screen high‐risk structural cases requiring priority verification in subsequent refinement procedures [17]. In essence, such crystallographic ML tasks are decision‐centric rather than merely prediction‐oriented.

Here, we develop a pre‐refinement machine‐learning framework for dopant site occupancy inference in M‐type barium and strontium hexaferrites. Our data‐driven model learns composition‐to‐occupancy statistical relationships directly from experimentally refined crystallographic records. It only takes host type, target‐dopant properties, target‐dopant amount, and co‐doping environment as inputs. Without diffraction patterns or pre‐assumed candidate atomic configurations, it can directly output candidate Wyckoff sites together with quantitative fractional occupancy distributions for dopants. Based on dopant‐level records extracted from reported refined crystal structures, we formulate the occupancy problem as a hierarchical workflow. To better align with realistic experimental use, we further introduce a reliability‐aware scheme that combines strong tabular baselines, training‐range assessment, and model‐disagreement analysis, allowing low‐confidence and boundary samples to be explicitly identified for priority refinement. The proposed framework not only provides a practical tool for refinement initialization and candidate screening, but also offers data‐driven insight into why dopant site occupancy in M‐type hexaferrites is strongly site‐dependent and cannot be reduced to a universal empirical rule.

## 2 Methods

### 2.1 Feature Representation and Data‐Splitting Strategy

As shown in Figure 1, a pre‐refinement machine‐learning workflow was developed to infer dopant site occupancy in M‐type hexaferrites. Features were constructed exclusively from pre‐refinement composition information [18, 19]. In this work, the term “pre‐refinement” refers to the condition in which the chemical composition of the target sample is known, while the site distribution of dopant elements remains undetermined. Specifically, pre‐refinement input information consists of host type, target‐dopant properties, target‐dopant amount, and co‐doping environment. No prior knowledge of dopant partitioning over individual crystallographic sites is required. For the dataset construction in the present study, all dopant amounts are extracted from Rietveld‐refined CIF files. Dataset details can be found in Note S1. These values represent real dopant contents within the crystal phase rather than nominal recipe concentrations. Refined compositional data were therefore used for both model training and external validation. For predictions on new materials, the experimentally measured incorporated dopant amount should be prioritized as model input. When only nominal recipe‐derived composition is available, it can be adopted as an approximate input to produce candidate occupancy hypotheses. Nevertheless, prediction uncertainty will increase if a substantial divergence exists between nominal composition and real incorporated composition.

**FIGURE 1:**
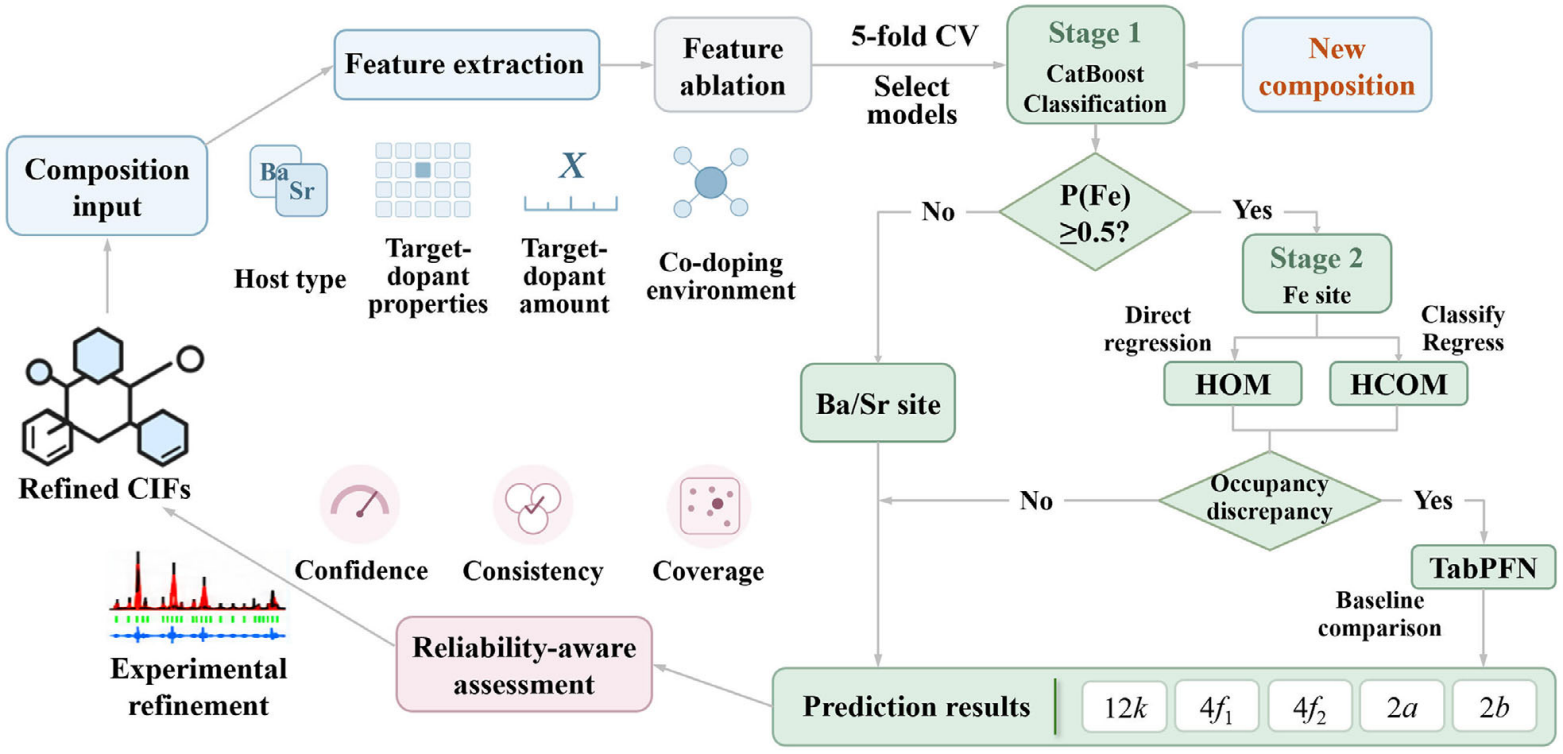
Workflow of the dopant site occupancy prediction framework for M‐type hexaferrites, mainly including dataset construction, feature engineering, hierarchical prediction, reliability‐aware assessment, and model optimization.

Target‐dopant properties covered periodic, valence‐related, size‐related, charge‐related, and bonding‐related properties [20, 21, 22]. To examine the contribution of different chemical information levels, feature selection was performed by grouped chemical ablation within the training set [23]. The external validation set was not involved in feature selection, model selection, or parameter tuning. The grouped ablation included target_host_16_, target_host_context_21_, weighted_physical_31_, weighted_core_37_, mean_weighted_core_45_, and full_89_ feature sets. It started from the basic target_host_16_ feature set, which contains target‐dopant properties and host type, and then progressively added target‐dopant amount, co‐doping environment, extended weighted co‐dopant environment, and finally the full candidate descriptor pool. The CatBoost classifier for Ba/Sr‐site vs. Fe‐sublattice discrimination adopts the target_host_16_ feature set, which consists of 16 input descriptors. By contrast, the Fe‐site occupancy prediction utilizes the weighted_physical_31_ feature set. As shown in Table S1, predictions for Sc and Mg site occupancy are generated using this weighted_physical_31_ feature set.

### 2.2 Hierarchical Occupancy‐Model Workflow

Site occupancy prediction was formulated as a hierarchical task. The first stage predicts whether the target dopant prefers the Ba/Sr sublattice or the Fe sublattice. Model training and prediction details can be found in Note S3. The Fe‐sublattice occupancy probability P(Fe) was used for this decision, with P(Fe) = 0.5 as the default threshold for Ba/Sr‐site vs. Fe‐sublattice classification. For this binary classification problem, several candidate models were compared, and CatBoost was selected as the final classifier based on grouped cross‐validation performance. The second stage is applied only to dopants assigned to the Fe sublattice and estimates fractional occupancies over the five inequivalent Fe sites, 12*k*, 4*f*_1_​, 4*f*_2_​, 2*a*, and 2*b*. Homogeneous occupancy‐model (HOM) screening was first performed as a uniform regression reference, in which the same regression algorithm was applied to the continuous occupancy outputs of all five Fe sites. Because Fe‐site occupancy contains both zero and nonzero values, heterogeneous cooperative occupancy‐model (HCOM) screening was adopted by combining site‐wise occupancy classification with occupancy‐amount regression. The candidate site‐occupancy classifiers included random forest, ExtraTrees, gradient boosting [24], XGBoost, LightGBM, and CatBoost. The candidate occupancy‐amount regressors included Huber regression [25], gradient boosting, XGBoost, LightGBM, and CatBoost. For each Fe site, the classifier determines whether occupancy is present, and the regressor estimates the corresponding occupancy amount. The final site occupancy is retained only when the predicted occupancy probability exceeds the decision threshold, otherwise it is set to zero. TabPFN was additionally introduced as an independent small‐sample baseline for robustness comparison [26]. When discrepancies exist between HOM and HCOM outputs, prediction results from the TabPFN baseline are referenced for validation.

To improve practical interpretability, the framework further incorporated a reliability‐aware assessment. Reliability was determined jointly from sublattice‐classification confidence, agreement between the main model and reference models, consistency with the TabPFN baseline, empirical training‐domain coverage, and whether the target dopant had been represented in the training set. On this basis, each prediction was assigned a reliability level and a refinement‐priority label. Low‐confidence predictions were treated as boundary or extrapolative cases requiring priority verification rather than as simple model failures.

## 3 Results and Discussion

### 3.1 Data‐Set and Validation Framework

In this work, dopant site occupancy in M‐type hexaferrites is formulated as a pre‐refinement prediction task. The curated dataset encompasses multidimensional structural information, including chemical composition, host type, target dopant, and refined site‐occupancy data. Each record represents one target dopant in a refined sample, while the remaining dopants in a co‐doped composition are encoded as its co‐doping environment. Figure 2a,b illustrates the workflow of crystallographic information file (CIF) screening, target row construction, and dataset division. Figure 2a summarizes the initial dataset construction and presents the target‐dopant distribution and partitioning of the Ba/Sr‐site records. While Figure 2b shows the weighted target‐dopant distribution and partitioning of the Fe‐sublattice records. A total of 496 CIFs were initially collected. After structural screening, 371 qualified crystal structures were retained and transformed into 494 dopant‐level records, comprising 369 Fe‐sublattice records and 125 Ba/Sr‐site records.

**FIGURE 2:**
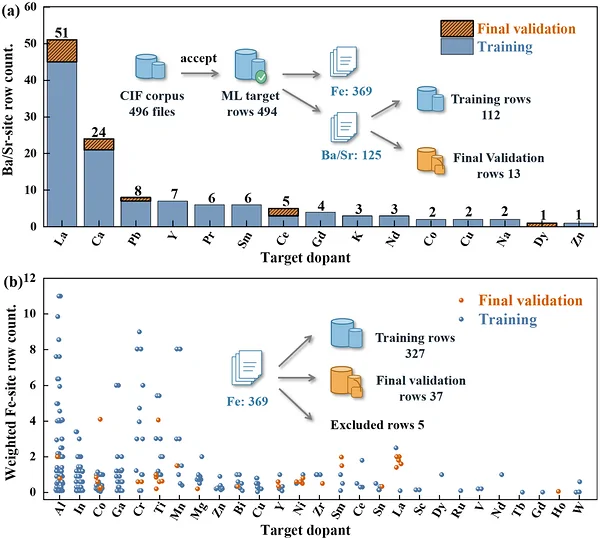
Distribution and splitting of target‐row records for dopant site occupancy prediction. (a) Ba/Sr‐site target‐row distribution for different target dopants. Ba/Sr‐site records were divided into 112 training rows and 13 final validation rows. (b) Weighted Fe‐site target‐row distribution for different target dopants. Fe‐site records were divided into 327 training rows, 37 final validation rows, and 5 excluded rows. Blue and orange marks denote training and final validation data.

The 494 dopant‐level records used in this work were extracted from refined crystal structures reported in the literature. Although most records correspond to structures refined at or near room temperature, the workflow is not limited to room temperature conditions. Sample preparation methods, synthesis conditions, and thermal history may affect the degree of cation ordering and the site‐dependent dopant occupancy. Since detailed preparation information is not uniformly, systematically, and reliably recorded across all literature sources, it was not explicitly included as input features in the present work. Instead, the current model primarily infers occupancy patterns based on the host type, target‐dopant properties, target‐dopant amount, and co‐doping environment. To a certain extent, these descriptors implicitly encode the underlying thermodynamic tendencies and the typical experimental context under which the reported structures were formed.

In addition, to prevent information leakage arising from repeated or closely related compositions, a composition‐level holdout strategy was adopted. Records with the same host type and normalized chemical formula were assigned to the same data subset. Of the 494 records, 439 were used for model training and cross‐validation, including 327 Fe‐sublattice records and 112 Ba/Sr‐site records. The external‐validation set contained 50 independent records from 31 material systems, comprising 37 Fe‐sublattice records and 13 Ba/Sr‐site records. The remaining five Fe‐sublattice records were excluded from model development and validation according to the data‐screening criteria. Compared with conventional random record splitting, this composition‐aware partitioning strategy provides a more rigorous assessment of model generalization to previously unseen compositions.

### 3.2 Hierarchical Site Occupancy Prediction

Dopant site occupancy prediction for M‐type hexaferrites is not a single‐label classification task, but a hybrid modeling task integrating binary sublattice assignment, sparse site activation, and continuous occupancy fractions. Such multi‐modal output space possesses definite crystallographic physical essence. Dopant atoms in M‐type hexaferrites follow a hierarchical occupation behavior, which prioritizes the occupancy competition between Ba/Sr sites and Fe sublattice at the first stage. Once dopants are thermodynamically inclined to incorporate into the Fe framework, they will further distribute across five crystallographically inequivalent Fe sites (12*k*, 4*f*_1_​, 4*f*_2_​, 2*a*, and 2*b*) with specific fractional occupancy values.

3.3

The first modeling stage focuses on binary sublattice discrimination, which aims to quantify the occupation propensity of target dopants toward Ba/Sr sites or Fe sublattice. For this sublattice screening task, the curated target_host_16_ feature set yields optimal performance via grouped cross‐validation on the training set. Figure 3c presents the performance loss of different feature groups relative to the best‐performing feature set. Feature group sets A‐I represent target_host_16_, target_host_context_21_, weighted_physical_31_, weighted_core_37_, mean_weighted_core_45_, no_min_max_std_57_, no_identity_mass_76_, no_extreme_std_41_, and full_89_. It is superior to the extended feature sets that included additional co‐dopant statistics and the full candidate descriptor pool. This demonstrates that intrinsic elemental properties of target dopants (including ionic radius, valence state, bonding affinity) and intrinsic matrix characteristics dominate the Ba/Sr‐Fe sublattice occupation competition. The blind feature expansion cannot bring performance improvement or extra chemical interpretability.

**FIGURE 3:**
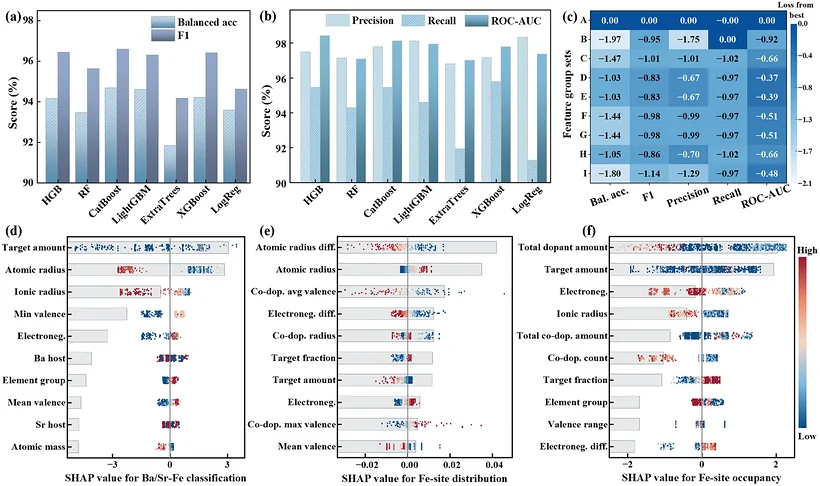
Model comparison, feature‐group ablation and post hoc feature interpretation. (a) Mean balanced accuracy and F1 of candidate classifiers for Ba/Sr–Fe sublattice classification. (b) Mean precision, recall, and ROC‐AUC of the classifiers. (c) Cross‐validation performance loss for feature‐set groups. SHAP analysis of (d) the CatBoost classifier for Ba/Sr‐vs.‐Fe sublattice classification, (e) the direct‐Huber model for normalized Fe‐site‐distribution interpretation, and (f) the XGBoost occupancy‐probability classifier within HCOM. Co‐dop., electroneg. and diff. denote co‐dopant, electronegativity, and the difference between target‐dopant amount and the amount‐weighted average co‐dopant value, respectively. Feature labels without Co‐dop. refer to target‐dopant descriptors by default.

As illustrated in Figure 3, we comprehensively compared multiple classifiers, including CatBoost, LightGBM, XGBoost, Random Forest, ExtraTrees, Histogram‐Based Gradient Boosting, and Logistic Regression. Under the same 16 input features and grouped 5‐fold cross‐validation, CatBoost achieved the best overall performance across all tested candidates. It delivers the highest mean balanced accuracy of 0.9469, together with mean F1‐score of 0.9660 and ROC‐AUC of 0.9812. Therefore, CatBoost was employed to distinguish between Ba/Sr‐site and Fe‐sublattice preference. Trained on 439 training records, this model correctly assigned 48 out of 50 external‐validation entries, yielding 96% classification accuracy. This external‐validation outcome further confirms the robustness of our model selection.

Based on SHAP, an interpretive analysis was conducted on the three levels of the hierarchical occupancy workflow. The mean‐absolute SHAP values for the key features presented in Figure 3d–f are summarized in Table S2. The mean absolute SHAP value reflects the average magnitude of the contribution of a given feature to the corresponding model output. As shown in Figure 3d, target‐dopant amount, atomic radius, and ionic radius are the dominant contributors in the Ba/Sr‐site vs. Fe‐sublattice classifier. Their respective mean‐absolute SHAP values are 1.7180, 1.6765, and 0.9961, respectively. These results indicate that dopant amount and size compatibility jointly govern sublattice selection between the Ba/Sr site and the Fe sublattice. Figure 3e further shows that target‐dopant radius, co‐dopant mean oxidation state, electronegativity, and co‐dopant radius have the largest effects on normalized Fe‐site allocation. This indicates that the relative distribution among the five Fe sites is controlled not only by the target dopant itself, but also by the charge and size environment introduced by co‐doping. Figure 3f shows that total dopant amount and target‐dopant amount are the most influential features in the HCOM occupancy‐probability component, followed by target electronegativity and target ionic radius. This indicates that whether a specific Fe site is activated is strongly related to the overall dopant loading, while the detailed site preference is further modulated by the chemical size and bonding characteristics of the target dopant.

### 3.4 Heterogeneous Cooperative Occupancy Model

Following the identification of the Ba/Sr and Fe sublattices, the target elements were further analyzed to determine their fractional occupancy distributions among the five inequivalent Fe sites, namely 12*k*, 4*f*_1_, 4*f*_2_, 2*a*, and 2*b*. Compared with sublattice identification, Fe‐site prediction requires not only accurate identification of the most preferred occupancy site for a given element but also quantitative characterization of weak occupancy, zero occupancy, and simultaneous occupancy across multiple sites. This task therefore provides a more stringent assessment of the model's ability to capture and predict local occupancy patterns in crystal structures.

As shown in Figure 4a, HOM screening was employed to compare the performance of different algorithms in predicting continuous Fe‐site occupancies. The Huber model achieved the lowest cross‐validation (CV) macro mean absolute error (macro MAE) of 0.0537 among all isomorphic models. Huber was therefore selected as the preferred regression model for predicting the continuous occupancies of the five Fe sites. Building upon this result, a HCOM model was further developed by separating Fe‐site occupancy identification from the estimation of positive occupancy values. The model first determines whether a given Fe site is likely to be occupied and subsequently estimates the corresponding fractional occupancy for sites predicted to exhibit occupancy. As shown in Figure 4b, the XGBoost‐Huber model achieved the lowest CV macro MAE among the various classification‐regression combinations and outperformed CatBoost‐Huber, XGBoost‐XGBoost, Random Forest‐Huber, and CatBoost‐Gradient Boosting. In the HCOM framework, each candidate consists of an occupied‐site classifier and an occupancy‐amount estimator. Repeated algorithm‐pair names in Figure 4b indicate that the same classifier‐estimator pair was evaluated using two output‐conversion rules. For duplicated algorithm‐pair names, the first occurrence first determines whether a site is occupied. The predicted occupancy amount is retained only when the site is classified as occupied, otherwise, the site is predicted as 0. The second occurrence does not directly set the site to 0, but instead calculates the occupancy amount as the product of the occupied‐site probability and the predicted occupancy amount. Compared with the single isomorphic Huber model, the XGBoost‐Huber model reduced the CV macro MAE from 0.0537 to 0.0509. These results demonstrate that the hierarchical collaborative modeling strategy, which integrates occupancy identification with quantitative occupancy estimation, can effectively address the challenges associated with zero occupancy and weak occupancy in Fe‐site prediction.

**FIGURE 4:**
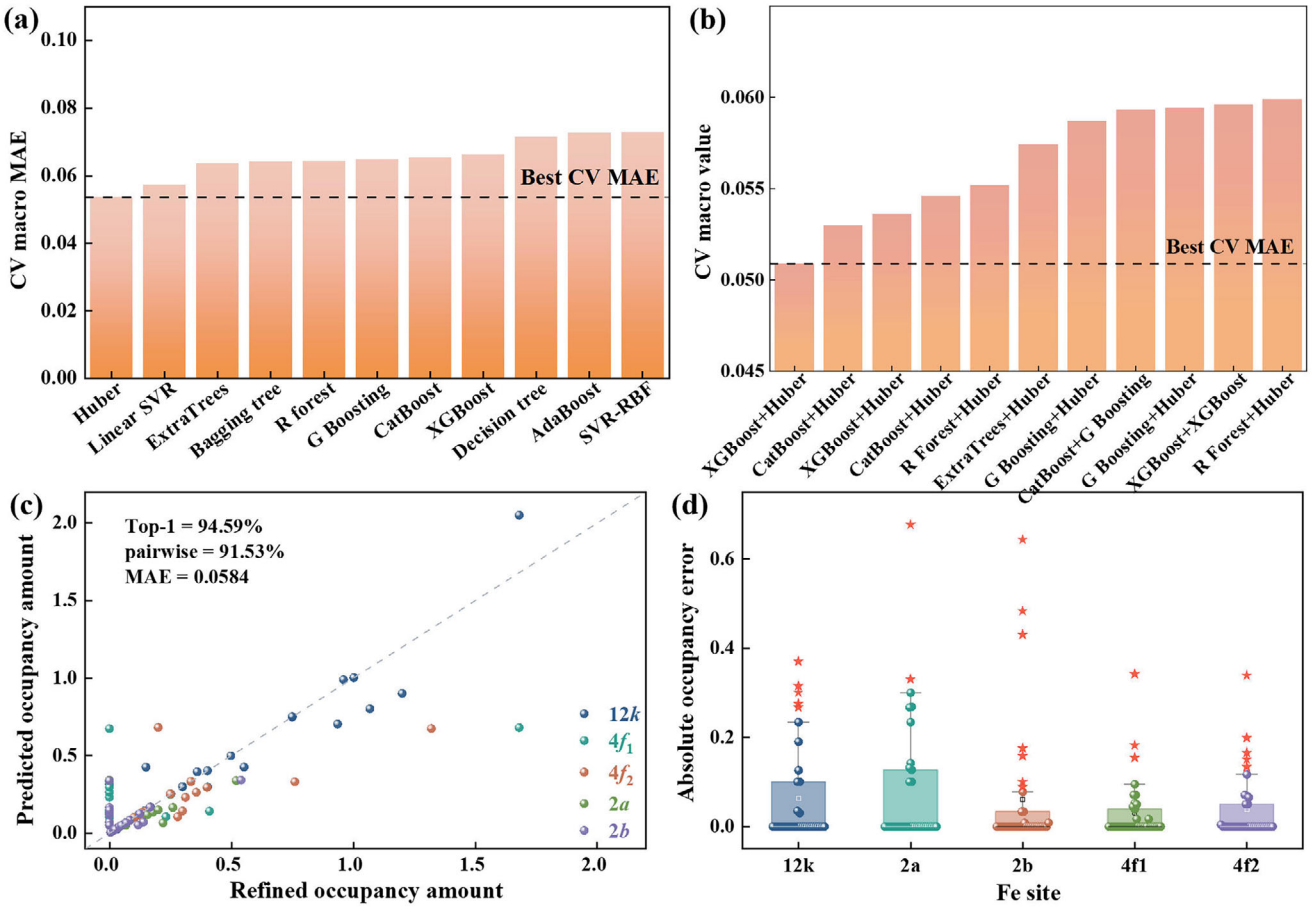
Fe‐site occupancy‐model screening and external validation. (a) Cross‐validated macro MAE for HOM candidates. (b) Cross‐validated metric values for HCOM candidates. The dashed line marks the best CV macro MAE. (c) External‐validation comparison between refined and predicted occupancy amounts. The dashed diagonal indicates equality. (d) Absolute occupancy‐error distributions for the five Fe sites.

The quantitative external‐validation results in Figure 4c demonstrate the good performance of the present model. Among the 50 external‐validation records, 48 records obtain correct sublattice assignments, achieving an accuracy of 96%. For the 37 Fe‐site external‐validation records, the model reaches a macro‐averaged MAE of 0.0584, alongside a top‐1 dominant‐site identification accuracy of 94.59%. The pairwise ranking accuracy is 91.53%. Top‐1 site accuracy measures whether the site with the largest predicted occupancy is the same as that with the largest experimental occupancy, while pairwise ranking accuracy measures the consistency between predicted and experimental orderings of the five Fe‐site occupancies. The predicted occupancies in the external validation set were generally distributed close to the diagonal relative to the refined occupancies, indicating that the model can reliably capture the fractional occupancy trends across the five Fe sites. Figure 4d presents the absolute prediction errors for the different Fe sites. The errors exhibited a non‐uniform distribution across the five sites, and a small number of outliers remained at certain sites. Therefore, the heterogeneous cooperative occupancy model improved the prediction of fractional occupancies across the five Fe sites while maintaining stable identification of the dominant occupancy site. Nevertheless, site‐dependent errors still require further verification during subsequent refinement in conjunction with specific samples.

To enable the model to output reliable occupancy hypotheses while also identifying samples that require priority manual review, we further integrated the sublattice‐discrimination result, Fe‐site main‐model prediction, CatBoost‐Huber reference model, TabPFN small‐sample baseline, and sample applicability‐domain information to assign confidence levels and refinement priorities [27]. As shown in Figure 5a, model confidence is closely related to the training‐experience domain. Samples within the training‐experience domain (in‐domain) are mostly assigned to the high‐confidence level. Samples at the boundary of the training‐experience domain (edge) mainly correspond to medium confidence. In contrast, samples from outside the training distribution (OOD) and far beyond the training distribution (extreme OOD) are more likely to be classified as having medium, low, or extremely low confidence. In this work, 44 out of 50 external‐validation records were assigned high or medium confidence, accounting for 88% of the validation set.

**FIGURE 5:**
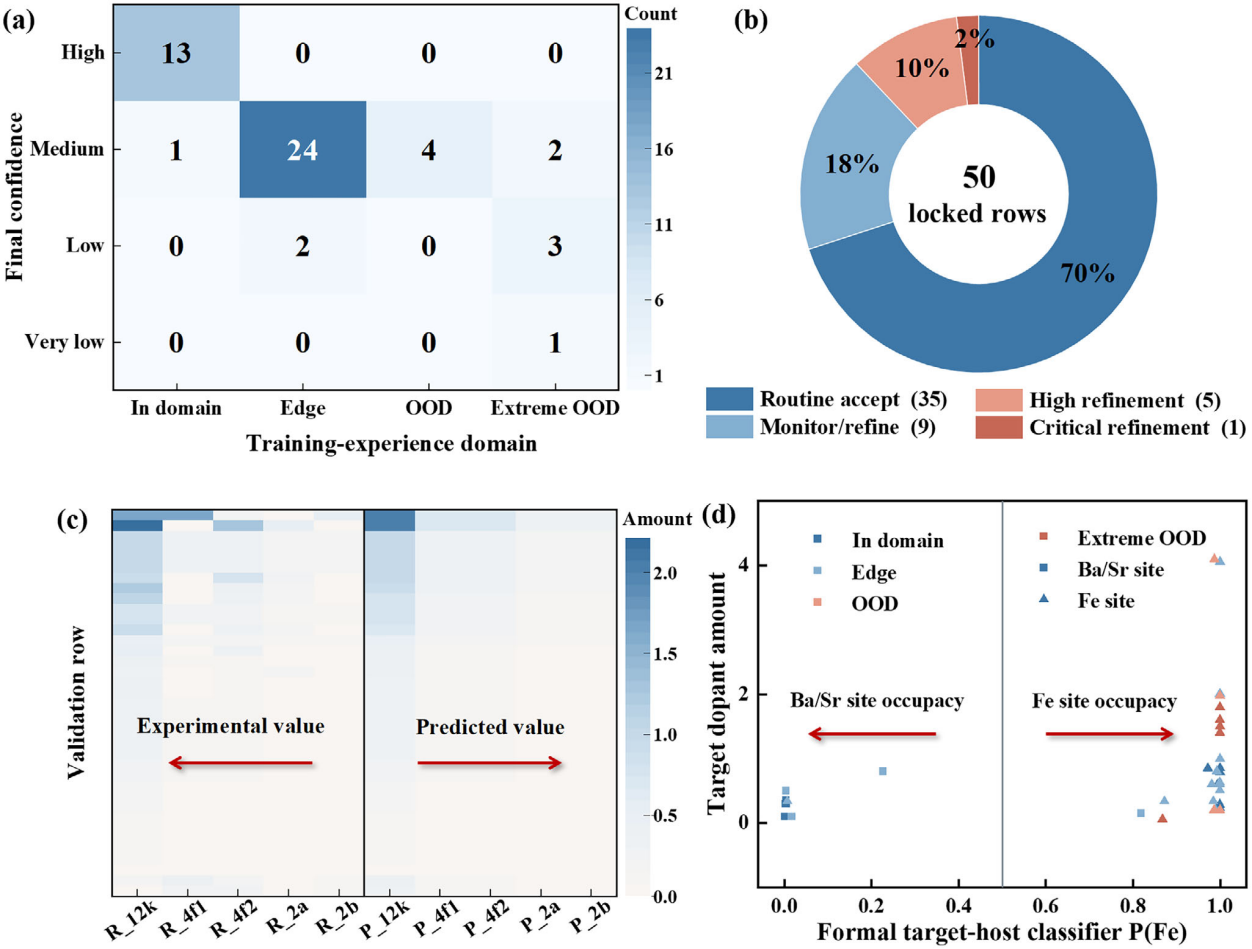
Reliability‐aware assessment of 50 external‐validation rows. (a) Relationship between the training‐experience domain and final confidence. (b) Distribution of refinement‐priority classes. (c) Refined (R) and predicted (P) occupancies across the five Fe sites for the validation rows. (d) Target‐dopant amount vs. Fe‐sublattice occupancy probability P(Fe).

Built upon dopant‐site occupancy predictions and reliability‐aware assessment, this framework assigns refinement‐priority labels to each sample. Figure 5b classifies the external‐validation samples into four refinement‐priority levels, including routine accept (directly usable as an initial model), monitor/refine (recommended for review), high refinement (high‐priority refinement), and critical refinement. It should be noted that low confidence does not indicate prediction failure. Rather, it provides a priority‐review signal for manual refinement, for which alternative sites, co‐doping competition effects, and abnormal occupancy distributions should be carefully examined. For high‐confidence samples within the training‐experience domain, the model can provide highly credible candidate occupancy hypotheses. For medium‐confidence samples, the prediction can serve as the main reference, but should still be reviewed together with the local coordination environment and refinement residual factors. Figure 5c further shows the distribution of external‐validation samples over the training‐experience domain and Fe‐site occupancy amounts, which helps identify samples that combine extrapolative characteristics with complex Fe‐site occupancy. Figure 5d shows the relationship between target‐dopant amount and the Fe‐sublattice occupancy probability P(Fe), where P(Fe) denotes the Fe‐sublattice occupancy probability output by the Ba/Sr‐Fe sublattice classification model. When P(Fe) ≥ 0.5, the dopant is judged to preferentially occupy the Fe sublattice. When P(Fe) < 0.5, the dopant is judged to preferentially occupy the Ba/Sr site. The closer P(Fe) is to 0.5, the stronger the occupancy competition between the Ba/Sr site and the Fe sublattice, and such samples should be carefully checked during crystallographic refinement. The classification results show that this model goes beyond single occupancy prediction and can quantify sample refinement priority to support subsequent experimental refinement.

### 3.5 Boundary Identification and Refinement‐Oriented Validation

In the external validation, a small number of disagreement cases were concentrated near the Ba/Sr‐Fe sublattice‐competition region and the boundary of the training‐experience domain. For example, site assignment for Sr_0.85_Co_0.6_Dy_0.15_Fe_11.4_O_19_ was evaluated via two sequential steps. Ba/Sr‐site vs. Fe‐sublattice classification, followed by fractional‐occupancy prediction over the inequivalent Fe sites. For Dy, HOM gave a tendency toward the Fe sublattice. However, both the HCOM and the TabPFN baseline assign Dy to the Sr site. According to the multi‐model voting and reliability‐assessment workflow, Dy was finally assigned to the Sr site rather than Fe sublattice, consistent with the database‐refined structural result [28]. For Co, which was assigned to the Fe sublattice, the model predicted occupancy amounts of 0.300, 0.100, 0.100, 0.050, and 0.050 at 12*k*, 4*f*_1_, 4*f*_2_, 2*a*, and 2*b* sites, respectively. These values correspond to relative fractions of 50.00 %, 16.67 %, 16.67 %, 8.33 %, and 8.33 % of the total Co content. The 12*k* site carries the dominant Co occupancy, which is consistent with database‐refined structural results. The value of these samples is mainly to identify systems for which alternative occupancy models should be retained in subsequent refinement. Notably, for BaW_0.06_Fe_11.94_O_19_, although W did not appear as a target element in the training set, the model still correctly assigned it to the Fe sublattice and predicted a low‐content, multi‐site dispersed occupancy pattern. As shown in Table 1, the five‐site fractional occupancies predicted by the main model are 0.030, 0.010, 0.010, 0.005, and 0.005, respectively, which are identical to the refined values. This indicates that, even when W was absent as a target element in the training set, the model could still accurately recover the main occupancy pattern of low‐content, dispersed W incorporation into the Fe sublattice.

**TABLE 1:** Refined and predicted dopant amounts per formula unit at the five Fe sites for boundary‐validation cases.

Sample	Screening method	12*k*	4*f*_1_	4*f*_2_	2*a*	2*b*
**BaW_0.06_Fe_11.94_O_19_**	Main‐model prediction	0.030	0.010	0.010	0.005	0.005
	Commercial database	0.030	0.010	0.010	0.005	0.005
**SrSc_1.602_Fe_10.398_O_19_**	Commercial database	1.068	0	0.356	0.178	0
	HOM prediction	0.805	0.265	0.264	0.134	0.134
	HOM prediction with Sr‐Sc control	1.074	0	0.354	0.174	0
	HCOM prediction with Sr‐Sc control	1.068	0	0.356	0.178	0
**SrMg_0.0498_Sc_1.7917_Fe_10.1598_O_19_**	Sc, neutron refinement at 300 K	0.718	0	1.016	0.057	0
	Mg, neutron refinement at 300 K	0.0498	0	0	0	0
	Sc, HCOM prediction	0.896	0.299	0.299	0.149	0.149
	Mg, HCOM prediction	0.025	0.008	0.008	0.004	0.004

The Sc‐doped Sr‐hexaferrites further reveal the influence of training experience on Fe‐site distribution prediction. For SrSc_1.602_Fe_10.398_O_19_ with Sc amount of 1.602, the refined Sc occupancies at the 12*k*, 4*f*_2_, and 2*a* sites are 1.068, 0.356, and 0.178, respectively. These values correspond to 66.67 %, 22.22 %, and 11.11 % of the total Sc dopant, confirming that the 12*k* site is the most populated Sc site at this composition. As shown in Table 1, the refined Sc occupancy at the 4*f*_1_ site for SrSc_1.602_Fe_10.398_O_19_ is zero. In this case, the HOM predicted Sc occupancies of 0.805, 0.265, 0.264, 0.134, and 0.134 at the 12*k*, 4*f*_1_, 4*f*_2_, 2*a*, and 2*b* sites, respectively. The corresponding macro MAE is 0.1596. This result indicates that, when the Sr‐Sc compositions were held out as a whole, the model lacked local training experience for high‐doped Sr‐Sc samples and was therefore more strongly affected by the occupancy pattern of Ba‐Sc samples in the training set, leading to small occupancy misallocations at the 4*f*_1_ and 2*b* sites. To test whether this bias arose from insufficient Sr‐Sc training experience, the neighboring SrSc_1.404_Fe_10.596_O_19_ and SrSc_1.8_Fe_10.2_O_19_ samples were returned to the training set, while only SrSc_1.602_Fe_10.398_O_19_ was retained as the test sample. Under this setting, the heterogeneous occupancy model predicted values of 1.068, 0, 0.356, 0.178, and 0, respectively. The first‐layer homogeneous model prediction became closer to the database refined values, approximately 1.074, 0, 0.354, 0.174, and 0, with a macro MAE of approximately 0.0024. At this point, the site order was 12*k* > 4*f*_2_> 2*a* > 4*f*_1_ = 2*b*, consistent with the three‐site occupancy pattern of the database test sample. This comparison shows that, after local Sr‐Sc training information was included, the model itself could effectively predict the three‐site occupancy pattern of Sr‐Sc. It also confirms that the prediction bias in the external validation mainly resulted from insufficient Sr‐Sc training experience. In this work, neutron powder diffraction was used to quantitatively resolve the crystallographic site occupancy of SrMg_0.0498_Sc_1.7917_Fe_10.1598_O_19_ at 300 K. The corresponding sample‐preparation details, atomic coordinates, and refined site occupancies can be found in Note S4, while the crystallographic parameters obtained from neutron powder diffraction at 300 K are summarized in Table S3. As shown in Table 1, the HCOM model correctly identifies 12*k* as the dominant occupied site for Mg, which is consistent with neutron diffraction results. For Sc, HCOM successfully identifies the occupied sites, including 12*k*, 4*f*_2_, and 2*a*. In addition, V. Dixit et al. used DFT to compare the relative energies and magnetic properties of different dopant configurations [29, 30]. Their studies clarified the effects of site distribution on the magnetic moment and magnetocrystalline anisotropy and provided an important reference for this study.

### 3.6 Model Scalability and Refinement Priority

The feature‐contribution results in Figure 3d–f show that sublattice discrimination and Fe‐site occupancy prediction rely on different chemical information. For Ba/Sr‐Fe sublattice discrimination, features related to target‐dopant size, valence state, and bonding characteristics exert dominant influence. From grouped‐SHAP analysis, the normalized collective contribution of this group of features reaches 0.713. Consistently, perturbing these key features degraded the balanced accuracy, F1‐score, and ROC‐AUC by 0.3613, 0.1126, and 0.3373, respectively. By comparison, target‐dopant amount, elemental‐period information, and host type make only secondary contributions. For a given sample, these analyses can further pinpoint which features push the prediction toward Ba/Sr‐site or Fe‐sublattice occupancy and quantify their relative magnitudes. By contrast, Fe‐site occupancy prediction depends more strongly on target‐dopant amount and co‐doping environment. Perturbing the target‐dopant amount increases macro MAE by 0.0377, while perturbing the co‐doping environment increases macro MAE by 0.0331. Such quantitative metrics cannot be deduced from qualitative crystal‐chemical heuristics alone. Fe‐site preference is not controlled by a single descriptor. The 12*k*, 4*f*_1_, 4*f*_2_, and 2*a* sites are jointly affected by co‐doping environment, target‐dopant size, valence, and bonding properties, and target‐dopant amount. Meanwhile, the occupancy behavior of the 2*b* site exhibits heightened sensitivity to the host type. These findings rationalize why disparate experimental studies can report divergent preferred dopant sites for nominally identical hexaferrite systems.

In M‐type hexaferrites, the five Fe sites are not magnetically equivalent. 12*k*, 2*a* and 2*b* are spin‐up sublattices, whereas 4*f*_1_ and 4*f*_2_ are spin‐down. Consequently, a non‐magnetic dopant occupying spin‐down 4*f*_1_/4*f*_2_ sites increases the net magnetization, while occupation of spin‐up sites decreases it. The trigonal‐bipyramidal 2*b* site is a principal source of uniaxial magnetocrystalline anisotropy, so its occupation strongly affects the anisotropy field and coercivity. Hence, the magnetic effect induced by dopant elements strongly depends on whether dopants occupy the Ba/Sr sublattice or the Fe sublattice. Once dopants are incorporated into the Fe sublattice, their specific distribution over the five inequivalent Fe sites further exerts a substantial influence on the local coordination environment, superexchange pathways, and corresponding magnetic contributions. For dopants predicted to occupy the Fe sublattice, our workflow further outputs fractional occupancy distributions among the 12*k*, 4*f*_1_, 4*f*_2_, 2*a*, and 2*b* sites. As shown in Figure S1, the corresponding predictions can identify the magnetic sites primarily affected by the target dopant and provide structural information for analyzing changes in the net magnetic moment, superexchange interactions, and magnetic anisotropy.

Based on these results, we further carried out prospective screening of site occupancy for a broad set of rare‐earth (RE) dopants. The screening set covered Sc, Y, and the lanthanide elements, excluding Pm because it has no stable isotope [31, 32]. We have performed prospective predictions for rare‐earth dopants in BaFe_12_O_19_ and SrFe_12_O_19_ across three doping levels (*x* = 0.1, 0.2 and 0.3). Here, *x* denotes the nominal target‐dopant amount per formula unit and does not presuppose whether the dopant occupies the Ba/Sr site or the Fe sublattice. The Fe‐sublattice occupancy probability P(Fe) represents the model probability that the dopant enters the Fe sublattice rather than the Ba/Sr site. Figure 6 summarizes pre‐refinement occupancy predictions for 96 host‐dopant combinations, covering 16 rare‐earth elements in M‐type barium and strontium hexaferrites at three doping levels. Among these 96 predictions, 71 cases favor 2*d* site occupation, while 25 cases favor Fe‐sublattice occupation. At low doping levels, most rare‐earth dopants tend to occupy the 2*d* site. When the dopant concentration rises, the number of cases favoring Fe‐sublattice occupation increases substantially.

**FIGURE 6:**
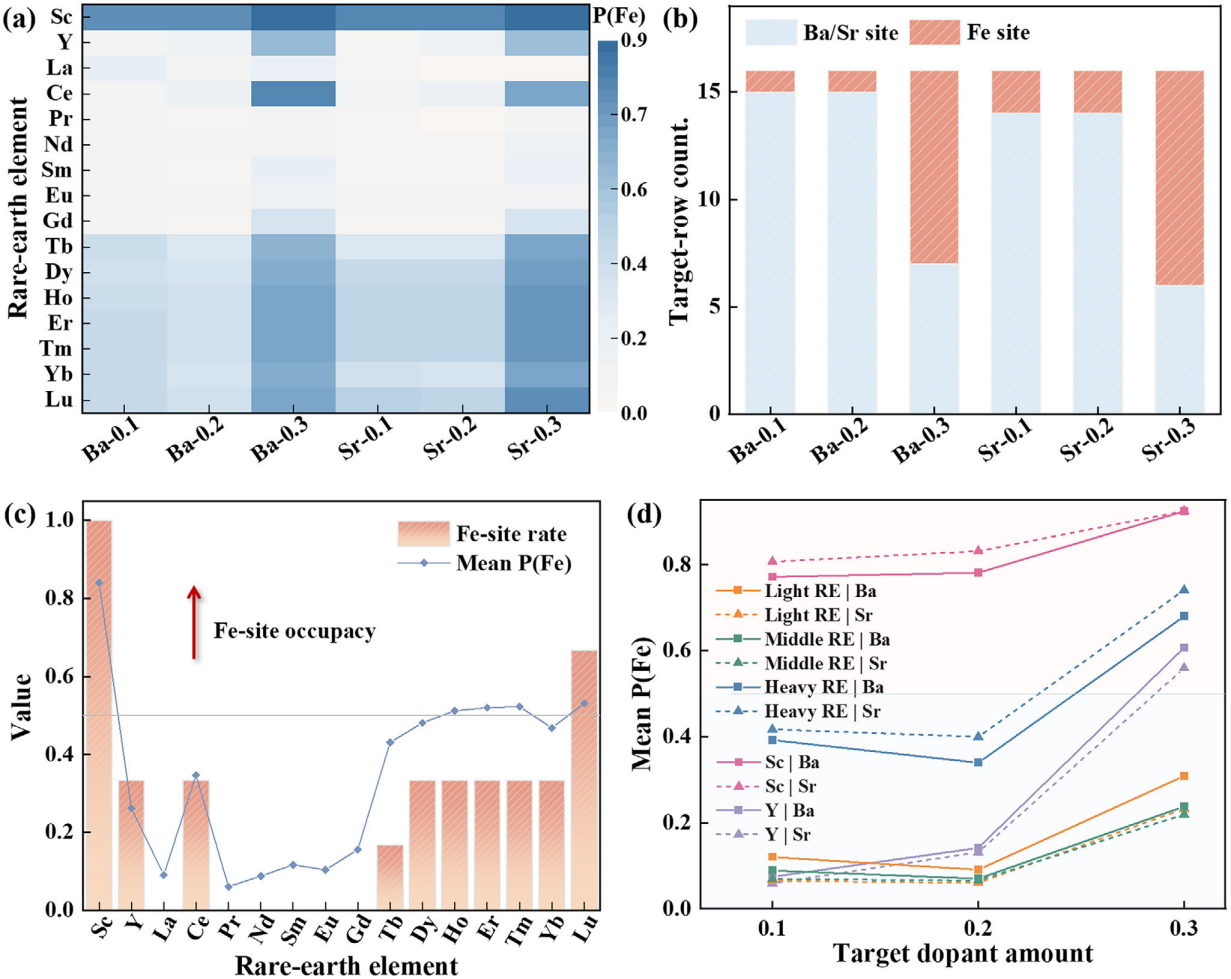
Pre‐refinement prospective screening of rare‐earth dopants in M‐type hexaferrites. (a) Fe‐sublattice occupancy probability P(Fe) as a function of dopant element, host type, and dopant content. (b) Entry counts for Ba/Sr‐site and Fe‐site preference for six host‐amount combinations. (c) Fe‐site rate and mean P(Fe) grouped by dopant. (d) Mean P(Fe) vs. target‐dopant amount in M‐type hexaferrites. The horizontal line denotes P(Fe) = 0.5.

Figure 6c gives the Fe‐site rate and mean P(Fe) for each rare‐earth element. The Fe‐site rate denotes the fraction of conditions satisfying P(Fe) ≥ 0.5, and mean P(Fe) denotes the average Fe‐sublattice occupancy probability of that element across the different host‐dopant combinations. The Fe‐sublattice occupancy probability does not follow a simple monotonic trend vs. atomic number or ionic size. For instance, light rare‐earth elements including La, Pr, and Nd generally maintain low P(Fe). By contrast, Sc exhibits a stable Fe‐sublattice preference across both barium and strontium hexaferrites. Notably, a given rare‐earth element can switch its dominant sublattice preference upon changing dopant concentration (Figure 6d). For several middle‐heavy lanthanides, higher dopant loading elevates P(Fe), which drives the dopant from Ba/Sr site preference toward Fe sublattice preference. Moreover, for Y and several middle‐to‐heavy lanthanides, the critical threshold of P(Fe) is reached at distinct doping concentrations for Ba‐host and Sr‐host hexaferrites. This means that the sublattice‐competition threshold is host‐dependent even for the identical dopant.

For occupancy‐sensitive doped systems, the present model can provide candidate sites, quantitative occupancy distributions, and reliability assessments without requiring a predefined site distribution. These quantitative results can serve as a site‐specific basis for analyzing differences in structural stability and magnetic properties among different occupancy configurations. For instance, the Ba‐Sc system is consistently predicted to prefer the 12*k* site across the doping range. This renders the Ba‐Sc system a high‐priority experimental candidate for exploring the mechanism of rare‐earth substitution within the Fe sublattice. The Ba‐Pr, Sr‐Pr, Sr‐Nd, Sr‐Sm, and Sr‐La systems exhibit 2*d* site occupation over multiple doping levels and can act as high‐confidence control systems for 2*d* site doping modification. By contrast, systems including Ba‐Dy, Ba‐Ho, Ba‐Tb, Ba‐Gd, Ba‐Y, Sr‐Sc, Sr‐Dy, Sr‐Ho, Sr‐Tb, Sr‐Ce, and Sr‐Y fall within the sublattice‐competition region and deserve focused structural‐refinement examination. Eu‐, Er‐, Tm‐, Yb‐, and Lu‐doped systems are repeatedly marked as OOD cases or low‐confidence predictions, making them more suitable as exploratory validation targets.

For these prioritized systems, the machine‐learning model is first used to screen likely candidate sites and estimate the corresponding occupancy distribution, substantially reducing the configurational space for subsequent energy calculations. In this sense, data‐driven screening and formation‐energy analysis can work together to support the study of dopant site occupancy and concentration effects. The present hierarchical prediction framework is conceptually transferable to other material systems that exhibit hierarchical site preferences, partial occupancies, and multi‐site distributions, such as spinels and garnets. Specifically, provided that a sufficient volume of structurally refined data is available, this methodology has great potential to be extended to other technologically important multi‐site materials.

## 4 Conclusion

In this work, we developed a pre‐refinement hierarchical machine‐learning workflow for dopant site occupancy prediction in M‐type barium and strontium hexaferrites using only composition‐derived information. The framework predicts Ba/Sr‐site vs. Fe‐sublattice preference, Fe‐site occupancy distribution, and prediction reliability, without requiring a predefined site‐distribution as input. The workflow combines three key features. A hierarchical prediction strategy, a reliability‐aware assessment for identifying low‐confidence and boundary samples, and data‐driven interpretation based on feature‐group ablation, perturbation, and SHAP analyses. The results show that sublattice discrimination is highly reliable, reaching 96% accuracy in consensus‐based external validation. In contrast, Fe‐site occupancy prediction provides quantitatively reasonable distributions and preserves dominant‐site identification for most cases. Feature analysis further indicates that sublattice assignment is mainly controlled by target‐dopant descriptors related to size, valence, and bonding characteristics. Whereas Fe‐site occupancy depends more strongly on target‐dopant amount and co‐doping environment. This framework provides a practical pre‐refinement screening and decision‐support tool for occupancy assignment in M‐type hexaferrites. Future improvements will benefit from more high‐quality refined structures and experimental feedback, particularly for complex co‐doped and extrapolative systems.

## Author Contributions

**Dan Liu**: conceptualization, supervision, project administration, funding acquisition, Writing – review and editing. **Jianyu Ren**: methodology, software, data curation, formal analysis, validation, visualization, writing – original draft, writing – review and editing. **Ruoshui Liu**: investigation, resources, writing – review and editing. **Yinong Yin**: investigation, data curation, validation. **Jianfeng Xi**: supervision, resources, writing – review and editing. **Xin Ming**: investigation, resources, writing – review and editing. **Jiazheng Hao**: investigation, validation, resources. **Tongyun Zhao**: resources, supervision, writing – review and editing. **Fengxia Hu**: resources, supervision, writing – review and editing. **Baogen Shen**: conceptualization, supervision, resources, funding acquisition, writing – review and editing.

## Conflicts of Interest

The authors declare no conflicts of interest.

## Supporting information




**Supporting File**: advs78493‐sup‐0001‐SuppMat.docx.

## Data Availability

The data that support the findings of this study are available from the corresponding author upon reasonable request.
