# Supplementary material for: Pre‐Refinement Prediction of Dopant Site Occupancy in M‐Type Hexaferrites via Hierarchical and Uncertainty‐Aware Machine‐Learning
Source: Adv Sci (Weinh). 2026 Oct 29:e78493. Online ahead of print. doi: 10.1002/advs.78493 (PMC13720265; doi:10.1002/advs.78493)
Supplement: Supplementary file 1 [file ADVS-9999-e78493-s001.docx]

**Supporting** **Information**

Dan Liu^1*^, Jianyu Ren^1^, Ruoshui Liu^2,3^, Yinong Yin^1^, Jianfeng Xi^1^, Xin Ming^3^, Jiazheng Hao^4^, Tongyun Zhao^5^, Fengxia Hu^5^, Baogen Shen^3,5^

1 Department of Physics, Beijing Technology and Business University, Beijing 100048, China

2 Ningbo Institute of Materials Technology and Engineering, Chinese Academy of Sciences, Ningbo 315201, China

3 Feicui Lake Laboratory, School of Materials Science and Engineering, Anhui University, Hefei 230601, China

4 Spallation Neutron Source Science Center (SNSSC), Dongguan 523803, China

5 State Key Laboratory of Magnetism, Institute of Physics, Chinese Academy of Sciences, Beijing 100190, China

**Note S1. Commercial database sources**

The database-refined reference occupancies used for comparison in Table 1 of the main text were obtained from two commercial crystallographic databases, PDF-5+ (International Centre for Diffraction Data, ICDD; https://www.icdd.com/pdf-5/) and SpringerMaterials (Springer Nature; https://materials.springer.com/). The database entries were screened for M-type hexaferrites containing the corresponding target dopants, and the reported/refined site occupancies were extracted from the database-refined structures. These occupancies were then converted into fractional occupancies over the five inequivalent Fe sites, 12*k*, 4*f*_1_, 4*f*_2_, 2*a*, and 2*b*, to enable direct comparison with the machine-learning predictions in the boundary-validation cases. The database-derived values are reported as reference occupancies in Table 1 of the main text.

**Note S2. Feature descriptors for Fe-site occupancy prediction**

Table S1 The weighted_physical_31_ feature set adopted for Fe‑site occupancy prediction. It contains 31 descriptors covering host type, target-dopant properties, target-dopant amount, and co-doping environment.

| **Feature category** | **Specific features** |
| --- | --- |
| **Host type** | Indicator variables for Ba, Sr, and mixed Ba/Sr hosts |
| **Target-dopant properties** | Period, group, electronegativity, atomic radius, mean ionic radius, mean oxidation state, minimum oxidation state, maximum oxidation state, oxidation-state range, and number of outer-shell electrons |
| **Target-dopant amount** | Total amount of the target dopant |
| **Co-doping environment** | Number of co-dopant species, total co-dopant amount, total amount of all dopants, fraction of the total dopant amount contributed by the target dopant, and an indicator variable for whether the system contains only one dopant species, amount-weighted means of co-dopant electronegativity, atomic radius, mean ionic radius, mean oxidation state, minimum oxidation state, maximum oxidation state, oxidation-state range, and number of outer-shell electrons, differences between the target-dopant value and the amount-weighted mean co-dopant value for electronegativity, atomic radius, mean ionic radius, and mean oxidation state |

**Note S3.** **Model training and prediction details**

Each modeling record corresponds to one target dopant in a refined sample, with the remaining dopants used to describe the co-doping environment. Sublattice labels were assigned from the refined structures, with the Fe sublattice encoded as 1 and the Ba/Sr site as 0. The regression targets were the site-resolved dopant amounts per formula unit. Training records were excluded if conflicting sublattice labels were found for the same host, composition, and target dopant, or if they failed the stoichiometric checks. These checks used reference totals of 1, 12, and 19 per formula unit for the Ba/Sr site, Fe sublattice, and oxygen, respectively, with permitted absolute deviations of 0.10, 0.20, and 0.30.

Sublattice classification used the target_host_16_ feature set, comprising the target-dopant amount, 12 elemental descriptors, and 3 indicator variables for Ba, Sr, and mixed Ba/Sr hosts. The elemental descriptors include atomic number, period, group, electronegativity, atomic radius, atomic mass, mean ionic radius, mean oxidation states, minimum oxidation states, maximum oxidation states, and range of oxidation states, and the number of outer-shell electrons. Fe-site prediction used the weighted_physical_31_ feature set, whose retained identifier denotes a set containing 31 features. These comprised the target-dopant amount, the above elemental descriptors excluding atomic number and atomic mass, 3 host indicators, 5 co-doping-environment descriptors, 8 amount-weighted co-dopant-property descriptors, and 4 descriptors representing differences between the target dopant and the co-dopants.

Training and validation records were grouped by host type and chemical formula, with no group shared between the training and external-validation sets. Model development used 439 training records, and external validation used 50 predefined records. Five-fold GroupKFold cross-validation was performed within the training set. Candidate classifiers were evaluated using identical splits and selected according to mean balanced accuracy. Missing numerical values were imputed using medians calculated from the training data for each fit, and standardization was additionally applied for Huber regression. Training records were weighted inversely by both the number of dopants in each CIF and the number of distinct CIF records sharing the same host, composition, and target dopant, to reduce the influence of repeated records. The external-validation set was not used for feature selection or model selection.

The final sublattice classifier was CatBoost. HOM used Huber regression, whereas HCOM combined XGBoost site-occupancy classifiers with Huber occupancy-amount regressors. For each Fe site in HCOM, an occupancy amount greater than 10⁻^9^ was labeled as nonzero occupancy, and the regressor was fitted only to records with nonzero occupancy at that site. During prediction, separate thresholds of 0.5 were applied to the sublattice-classification probability and the individual Fe-site occupancy probabilities. Negative predicted occupancy amounts were set to zero. Cross-validation metrics were averaged over the five folds, whereas external-validation metrics were calculated separately from the final model predictions on the fixed validation set.

The table below lists the three features with the highest mean absolute SHAP values in each panel of Fig. 3(d-f), quantifying their contributions to the corresponding model outputs.

Table S2 Mean absolute SHAP values of the top three ranked features for each panel in Fig. 3(d-f). Mean absolute SHAP values represent the average magnitude of feature contributions to the corresponding model output. Values for Fig. 3(d) are averaged over the analyzed samples, whereas those for Fig. 3(e, f) are averaged over the analyzed samples and the five Fe sites.

| **Panel** | **Features** | **Mean absolute SHAP value** |
| --- | --- | --- |
| Fig. 3(d) | Target amount | 1.7180 |
|  | Atomic radius of target dopant | 1.6765 |
|  | Ionic radius of target dopant | 0.9961 |
| Fig. 3(e) | Atomic radius difference between the target dopant and co-doping environment | 0.001098 |
|  | Atomic radius of target dopant | 0.000998 |
|  | Average valence state of the co-doped elements | 0.000741 |
| Fig. 3(f) | Total dopant amount | 0.7891 |
|  | Target amount | 0.7715 |
|  | Electronegativity of target dopant | 0.4032 |

**Note S4.** **Sample preparation and 300 K neutron refinement**

The starting materials were analytical grade SrCO_3_ (99.95%), Fe_2_O_3_ (99.95%), Sc_2_O_3_ (99.90%), and MgO (99.95%). Strontium ferrites with the composition SrFe_11.95-x_Sc_x_Mg_0.05_O_19_ were prepared by a solid-state reaction method. Stoichiometric mixtures of the oxides were thoroughly ground by ball milling and then calcined in corundum crucibles in air at 1300 °C for 1 h. The actual composition SrMg_0.0498_Sc_1.7917_Fe_10.1598_O_19_ was derived from the 300 K neutron diffraction refinement CIF, calculated using the site occupancies, crystallographic multiplicities, and Z value listed in Table S3. The refined total dopant amounts were used for model prediction, whereas the refined site-resolved occupancies were used for comparison with the predictions.

Table S3 The crystal structure parameters obtained by powder neutron diffraction at 300 K for the SrMg_0.0498_Sc_1.7917_Fe_10.1598_O_19_ solid solution.

| Atom | Wyckoff position | x | y | z | Site occupancy | Uiso / Å² |
| --- | --- | --- | --- | --- | --- | --- |
| Sr | 2*d* | 0.33333 | 0.66667 | 0.75000 | 1.000 | 0.076000 |
| Fe1/Sc1/Mg1 | 12*k* | 0.1681(7) | 0.3363(7) | 0.60943(16) | 0.8720/0.1197/0.0083 | 0.013600 |
| Fe2 | 4*f*_1_ | 0.333333 | 0.666667 | 0.0266(5) | 1.0001 | 0.013600 |
| Fe3/Sc3 | 4*f*_2_ | 0.333333 | 0.666667 | 0.1897(4) | 0.4921/0.5082 | 0.013600 |
| Fe4 | 2*b* | 0.000000 | 0.000000 | 0.250000 | 1.0000 | 0.013600 |
| Fe5/Sc5 | 2*a* | 0.000000 | 0.000000 | 0.000000 | 0.9434/0.0571 | 0.013600 |
| O1 | 4*e* | 0.000000 | 0.000000 | 0.1571(7) | 1.0001 | 0.011000 |
| O2 | 4*f* | 0.333333 | 0.666667 | 0.5615(7) | 1.0001 | 0.011000 |
| O3 | 6*h* | 0.167(5) | 0.333(5) | 0.250000 | 0.9364 | 0.011000 |
| O4 | 12*k* | 0.151(3) | 0.301(3) | 0.0518(4) | 1.0000 | 0.011000 |
| O5 | 12*k* | 0.507(3) | 0.013(3) | 0.1501(4) | 1.0000 | 0.011000 |

Space group: P6_3_/mmc, *a* = *b* = 5.9129(4) Å, *c* = 23.361(3) Å, *V* = 707.331707 Å³, *Z* = 2.

For SrMg_0.0498_Sc_1.7917_Fe_10.1598_O_19_, Sc exhibits a pronounced site-selective occupation behavior. According to the refined atom-site parameters from the 300 K CIF file, Sc is mainly distributed at the 4*f*_2_ and 12*k* sites. After considering the crystallographic site multiplicities, approximately 56.73% of the Sc ions occupy the 4*f*_2_ site, followed by 40.09% at the 12*k* site, while only a small fraction occupies the 2*a* site, approximately 3.19%. Mg is located at the 12*k* site. Considering the total dopant content of Sc and Mg, approximately 55.19% of the dopant ions occupy the 4*f*_2_ site, 41.70% occupy the 12*k* site, and 3.10% occupy the 2*a* site. These results indicate that, in this sample, the nonmagnetic or weakly magnetic substituent ions preferentially occupy the 4*f*_2_ and 12*k* sublattices.

**Note S5. Predicted Fe-site occupancies for 25 rare-earth candidate compositions**

Fig. S1. Predicted Fe-site occupancies for 25 rare-earth candidate compositions. (a) Ba-based systems (n = 11). (b) Sr-based systems (n = 14). Colors from pale yellow to dark blue indicate increasing predicted dopant occupancy at the corresponding Fe sites.
